# Factors affecting the academic performance of dental students in Riyadh, Saudi Arabia: a cross-sectional study

**DOI:** 10.3389/fdmed.2026.1762194

**Published:** 2026-02-13

**Authors:** Sanjeev B. Khanagar, Abdulrahman Alfaraj, Mohammed Mana Algrne, Faisal Khaled Almutairi, Ibrahim Aljuryyed, Ahmed Binobaid

**Affiliations:** 1 Preventive Dental Science Department, College of Dentistry, King Saud bin Abdulaziz University for Health Sciences, Riyadh, Saudi Arabia; 2 King Abdullah International Medical Research Centre, Riyadh, Saudi Arabia; 3 Ministry of the National Guard Health Affairs, Riyadh, Saudi Arabia; 4 College of Dentistry, King Saud bin Abdulaziz University for Health Sciences, Riyadh, Saudi Arabia; 5 Department of Restorative and Prosthetic Dental Sciences, College of Dentistry, King Saud bin Abdulaziz University for Health Sciences, Riyadh, Saudi Arabia

**Keywords:** academic, achievements, success, dental students, education, excellence, factors, performance

## Abstract

**Background:**

Academic performance is a key indicator of a student's success in education and it forms the foundation for carrier in dentistry. Therefore, the aim of this research was to identify the factors associated with poor academic performance among dental students in Riyadh, Saudi Arabia.

**Methods:**

A cross-sectional analytical study was conducted among dental students from six colleges located in Riyadh, Saudi Arabia. A self-administered structured questionnaire was used to collect data. The validity of the questionnaire was assessed using Aiken's V test, with each item achieving a score of 0.90 or higher. Test-retest reliability showed an intraclass correlation coefficient of 0.82. Descriptive analysis was performed, and Chi-square tests were conducted to assess the significance of the findings. Further regression analysis was carried out to determine the odds associated with each significant finding.

**Results:**

A total of 400 dental students were invited to participate, of whom 318 agreed, resulting in a response rate of 79.5%. Among the participants, 136 (42.8%) reported a GPA below 4.25. Chi-square analysis revealed that male students 90 (53.6%), students who smoked 43 (55.1%), students lacking motivation 67 (58.8%), those with irregular attendance 70 (61.9%) and students perceiving English as a barrier 74 (59.7%) were more prevalent in the lower academic performance group (*p* = 0.01). Binomial logistic regression analysis indicated that male students [Adjusted Odds Ratio (AOR) = 2.10, 95% CI: 1.12–3.94, *p* = 0.02], irregular attendance (AOR = 2.50, 95% CI: 1.40–4.50, *p* = 0.002), and students perceiving English as a barrier (AOR = 2.80, 95% CI: 1.57–5.00, *p* = 0.01) were more likely to have a GPA below 4.25. Social interactions with family and friends (AOR = 1.80, 95% CI: 0.94–3.45, *p* = 0.08) also showed a trend toward increased likelihood, though not statistically significant. Conversely, students reporting good sleep quality (AOR = 0.50, 95% CI: 0.26–1.01, *p* = 0.056) and higher motivation (AOR = 0.40, 95% CI: 0.22–0.73, *p* = 0.03) were less likely to have a GPA below 4.25.

**Conclusion:**

These findings offer a clear, evidence-based roadmap for dental colleges to develop targeted and effective interventions that improve academic outcomes.

## Introduction

Academic performance serves as an indicator of a student's success in education, reflecting how effectively they have achieved both their short-term and long-term goals (1). This performance is evaluated using multiple criteria, including grades, test results, and the successful completion of academic milestones such as professional degrees. It is characterized by metrics such as grade point average (GPA), standardized test scores, and educational goals and achievements (2, 3). The academic performance of dental students is of great importance, as they serve as the foundation for their future careers in oral healthcare (3, 4). Academic excellence provides dental students with a strong foundation of knowledge and essential skills to address complex oral health issues, adapt to evolving technologies, and contribute to advancements in the field(5). Hence, achieving a high level of academic success throughout dental training is critically important.

Dentistry is recognized as a high-stress profession, beginning in dental school, which is perceived as a highly challenging and stressful learning environment (6, 7). Dental education requires a high degree of cognitive functioning, critical thinking, and manual dexterity, alongside a demanding curriculum, limited control over scheduled classes, and a rapid succession of high-stakes examinations—all of which may contribute to diminished academic performance (7, 8). Furthermore, this environment can adversely affect cognitive abilities, impair emotional regulation, and negatively influence executive functions. These factors are linked to the perseverance that ultimately determines the academic success or failure of students during their dental training (4, 6, 7). Previous studies have reported that dental students experience a tremendous amount of stress related to academic workload, clinical performance pressure, fear of failure, anxiety, poor sleep quality, and burnout, all of which have been associated with poor academic outcomes (8–13).

Academic success is considered a primary criterion for the employment of recent graduates and is linked to increased student productivity and an improved overall quality of life. Therefore, it is crucial to understand the factors that influence academic performance, as they can have significant implications for students’ career prospects (14, 15). Furthermore, students’ academic performance not only reflects individual achievement but is also commonly used as a measure of institutional performance.

In Saudi Arabia, the academic performance of dental students is measured by their GPA. This GPA accounts for 30% of the differentiation and nomination process for residency programs conducted by the Saudi Commission for Health Specialties (16). Although existing research addresses academic performance in dental education globally, there remains a gap in the literature regarding the factors contributing to poor academic performance among dental students in Saudi Arabia. Therefore, the aim of this research was to identify the factors associated with poor academic performance among dental students in Riyadh, Saudi Arabia. The objectives were to assess the association of academic year, gender, and family income with the responses.

## Materials and methods

### Research design

A cross-sectional analytical study was conducted after obtaining ethical approval from the Institutional Review Board of the King Abdullah International Medical Research Center (KAIMRC) in Riyadh, Saudi Arabia (IRB Approval Number: 00000106725; Study Number: NRR25/066/1; approved on 23 January 2025).

### Sample size estimation

The sample size was calculated based on findings documented in the literature (17). It was determined to achieve 90% power and a 95% confidence interval for a prevalence rate of 50% (18). Consequently, the required sample size for this study was 312 participants.

### Study population and sampling technique

There are six dental colleges located in Riyadh, Saudi Arabia, and all these institutions were part of this study. After obtaining permission from the college administration to collect data on campus, the study participants were approached during their college hours at their respective colleges. A simple random sampling method was employed to select a minimum of fifty-five dental students from the clinical years to meet the quota from each university, provided they met the eligibility criteria.

### Eligibility criteria

#### Inclusion criteria

Dental students who were in their clinical years and those who displayed willingness to participate and provided signed informed consent form were included.

#### Exclusion criteria

Dental students in preclinical years.

### Data collection tool

The data were collected using a self-administered structured questionnaire, developed after reviewing similar studies documented in the literature (17, 19–22).

The content validity of the questionnaire was assessed by five content experts, each holding either a master's degree or a Doctor of Philosophy in Dental Public Health, all with strong research backgrounds. Aiken's V test was used to measure the level of agreement among the experts regarding the questionnaire items, with every item achieving a score of 0.90 or higher, indicating a robust expert consensus. A pilot study was conducted with twenty students from the College of Dentistry at King Saud bin Abdulaziz University for Health Sciences to evaluate the clarity and reliability of the questionnaire. To determine test-retest reliability, data were collected from the same participants after a four-week interval. An intraclass correlation coefficient of 0.82 was obtained, demonstrating good reliability of the instrument. However, these data were not included in the final analysis.

This questionnaire was divided into two sections. Questions 1 through 4 gathered demographic information about the participants, while Questions 5 through 18 consisted of closed-ended questions related to academic, social and personal variables.

Dental universities in Saudi Arabia consider students with a cumulative score of 4.75 and above out of a 5 GPA to be eligible for first class honors, while students scoring between 4.25 and 4.75 receive second class honors. Based on this criterion, participants were categorized into (1) those with an overall average of 4.25 and above, classified as having good academic performance, and those with an average lower than 4.25, classified as having poor academic performance.

### Data collection

The data were obtained using a questionnaire distributed either as printed copies or online via Google Forms. Study participants were informed that no identifiable data would be collected, ensuring the complete protection of their personal information and confidentiality. The filled questionnaire forms were collected on the same day. The data collection process was scheduled over a period of three months, from February 1, 2025, to April 30, 2025.

### Data analysis

The data collected were transferred into SPSS Statistical Software version 29 (IBM Corporation, Armonk, NY, USA). Data cleaning was conducted prior to the transfer to SPSS Statistical Software for analysis. Descriptive analysis was performed, and Chi-square tests were conducted to assess the significance of the findings. Further Binomial regression analysis was carried out to determine the odds associated with each significant finding. A *p* value < 0.05 was considered statistically significant.

## Results

In the present study, a total of 400 dental students were invited to participate, of whom 318 agreed, resulting in a response rate of 79.5%. Among the participants, 150 (47.2%) were female and 168 (52.8%) were male. Regarding academic year, 92 (28.9%) were in their 5th year, 137 (43.1%) in their 6th year, and 89 (28%) were interns. Concerning family income, 40 (12.6%) reported earning less than 10,000 SAR, 147 (46.2%) between 10,000 and 20,000 SAR, and 131 (41.2%) more than 20,000 SAR. For the screening question about GPA, 136 (42.8%) participants indicated their GPA was below 4.25. The details are presented in (Table 1).

**Table 1:** Demographic details of the study participants.

Variables	Category	Frequency (*n*)	Percentage (%)
Gender	Female	150	47.20
	Male	168	52.80
Academic year	5th year	92	28.90
	6th year	137	43.10
	Intern	89	28.00
Family income	Less than 10,000 SAR	40	12.60
	10,000–20,000 SAR	147	46.20
	More than 20,000 SAR	131	41.20
GPA	4.25 and above	182	57.20
	Lower than 4.25	136	42.80

Chi-square analysis of demographic data revealed that a significant proportion of students with a GPA below 4.25–52 (56.5%) were in their fifth year. A statistically significant difference (*p* < 0.001) was observed in gender distribution, with male students 90(53.6%), having a lower GPA. Smoking status was another significant factor (*p* = 0.01), with smokers, 43 (55.1%), being more prevalent in the lower GPA group. Motivation to continue the course showed a significant association with academic performance; students lacking motivation, 67 (58.8%), were more frequently observed in the lower academic performance group (*p* = 0.01). Attendance patterns also had a significant impact, as students with irregular attendance, 70 (61.9%), predominated among lower GPA group (*p* = 0.01). Additionally, language barriers played a crucial role, with students perceiving English as a barrier—74 (59.7%)—significantly constituting the majority in the lower-performing group (*p* = 0.01). Lastly, clinical experience was significantly associated with academic performance; students reporting inadequate clinical experience, 66 (49.6%), were more frequently found in the lower GPA group (*p* = 0.03), as presented in (Table 2).

**Table 2:** Sociodemographic characteristics of the study participants according to the academic performance.

Question	Category	4.25 and above (182)		Less than 4.25 (136)		*p* value
		*n*	%	*n*	%	
What is your academic year?	5th year	40	43.50	52	56.50	0.01^a^
	6th year	83	60.60	54	39.40	
	Intern	59	66.30	30	33.70	
What is your gender?	Female	104	69.30	46	30.70	0.01^a^
	Male	78	46.40	90	53.60	
What is your family income?	10,000–20,000	82	55.80	65	44.20	0.86
	Less than 10,000	24	60.00	16	40.00	
	More than 20,000	76	58.00	55	42	
Do you live alone?	Yes	30	48.40	32	51.60	0.11
	No	152	59.40	104	40.60	
Do you smoke?	Yes	35	44.90	43	55.10	0.01^a^
	No	147	61.30	93	38.80	
Do you have good sleep quality?	Yes	70	60.90	45	39.10	0.32
	No	112	55.20	91	44.80	
Was dentistry course your first choice for graduation?	Yes	49	58.30	35	41.70	0.81
	No	133	56.80	101	43.20	
Do you work in any part time job after the college hours?	Yes	29	53.70	25	46.30	0.56
	No	153	58.00	111	42.00	
Do you have difficulty in managing your time?	Yes	120	56.10	94	43.90	0.54
	No	62	59.60	42	40.40	
Do you have the motivation to continue in the course?	Yes	135	66.20	69	33.80	0.01
	No	47	41.20	67	58.80	
Do you feel satisfied with your education environment?	Yes	75	57.30	56	42.70	0.99
	No	107	57.20	80	42.80	
Do you show irregular attendance in the college?	Yes	43	38.10	70	61.90	0.01^a^
	No	139	67.80	66	32.20	
Does the quality of teaching in your college affect your performance?	Yes	143	58.80	100	41.20	0.29
	No	39	52.00	36	48.00	
Does the faculty-student relationship in the college affect your performance?	Yes	144	59.30	99	40.70	0.18
	No	38	50.70	37	49.30	
Is English a language barrier to your academic performance?	Yes	50	40.30	74	59.70	0.01^a^
	No	132	68.00	62	32.00	
Do you have access to educational resources?	Yes	113	54.90	93	45.10	0.24
	No	69	61.60	43	38.40	
Do you have adequate clinical experience?	Yes	115	62.20	70	37.80	0.03
	No	67	50.40	66	49.60	
Do you dedicate a substantial amount of time to chatting with your family/friends?	Yes	97	53.00	86	47.00	0.07
	No	85	63.00	50	37.00	

^a^ Statistical significance was set at 0.05; *n* represents the number of samples.

Binomial logistic regression identified several significant and marginally significant predictors associated with poor academic performance (GPA < 4.25). The following confounders were adjusted for in the analysis of the variables against each of the significant independent variable: gender, academic year, smoking, motivation to continue the course, irregular attendance, English as a language barrier. Among these predictors, gender played a crucial role, with male students being 2.1 times more likely to have a GPA below 4.25 compared to female students [Adjusted Odds Ratio (AOR) = 2.10, 95% CI: 1.12–3.93, *p* = 0.02]. Motivation to continue the course was another strong predictor; students with higher motivation were less likely to have a GPA below 4.25 (AOR = 0.40, 95% CI: 0.22–0.73, *p* = 0.03). Irregular attendance also emerged as a significant predictor, with students who frequently missed classes being 2.5 times more likely to have poor academic performance (AOR = 2.50, 95% CI: 1.40–4.50, *p* = 0.002). Furthermore, English language difficulties significantly impacted academic performance, with students perceiving English as a barrier being 2.8 times more likely to have a GPA below 4.25 (AOR = 2.80, 95% CI: 1.57–5.00, *p* < 0.01). Additionally, students in the internship year were less likely to have a GPA below 4.25 compared to fifth-year students (AOR = 0.45, 95% CI: 0.21–0.97, *p* = 0.04).

Apart from these significant predictors, some marginally significant factors were also identified that may influence academic performance. Sleep quality emerged as an important factor, with students reporting good sleep quality being less likely to have a GPA below 4.25 (AOR = 0.50, 95% CI: 0.26–1.01, *p* = 0.05). Clinical experience also showed a marginally protective effect; students who perceived their clinical exposure as adequate were less likely to have poor academic performance (AOR = 0.55, 95% CI: 0.29–1.04, *p* = 0.06). Conversely, time dedicated to social interactions with family and friends showed a trend toward increasing the likelihood of poor performance, with students engaging more in social activities being 1.8 times more likely to have a GPA below 4.25 (AOR = 1.80, 95% CI: 0.94–3.45, *p* = 0.08), as shown in (Table 3).

**Table 3:** Binomial regression analysis for factors associated with poor academic performance among undergraduate dental students.

Variable	GPA lower than 4.25					
	OR (Unadjusted)	95% CI (Unadjusted)	*p*-value (Unadjusted)	OR (Adjusted)	95% CI (Adjusted)	*p*-value (Adjusted)
Gender (Male vs. Female)	2.46	(1.331,4.553)	0.01^a^	2.10	(1.120,3.935)	0.02^a^
Academic Year (6th vs. 5th Year)	0.51	(0.274,0.984)	0.04^a^	0.60	(0.312,1.150)	0.12
Academic Year (Intern vs.5th Year)	0.40	(0.181,0.885)	0.01^a^	0.45	(0.210,0.970)	0.01^a^
Smoking (Yes vs. No)	1.33	(0.690,2.561)	0.40	1.20	(0.620,2.330)	0.58
Good Sleep Quality (Yes vs. No)	0.55	(0.287,1.059)	0.06	0.50	(0.260,1.010)	0.05
Motivation to Continue Course (Yes vs. No)	0.38	(0.211,0.704)	0.01^a^	0.40	(0.220,0.730)	0.01^a^
Irregular Attendance (Yes vs. No)	2.75	(1.533,4.967)	0.01^a^	2.50	(1.400,4.500)	0.01^a^
English as a Barrier (Yes vs. No)	3.00	(1.703,5.316)	0.01^a^	2.80	(1.570,5.000)	0.01^a^
Time Dedicated to Family/Friends (Yes vs. No)	1.97	(0.982,3.968)	0.05	1.80	(0.900,3.600)	0.08

^a^ Statistical significance set at 0.05, OR, odds ratio; CI, confidence interval.

Overall, these findings underscore the importance of factors such as attendance, language proficiency, motivation, and structured academic engagement in influencing student performance. While gender and language barriers present significant challenges, positive factors like sufficient sleep, adequate clinical exposure, and strong motivation may serve as protective elements against poor academic outcomes.

## Discussion

Dentistry is regarded as a profession with high levels of stress, beginning during dental school, which is widely recognized as a highly challenging and demanding learning environment. This primarily encompasses the substantial workload, patient management, and rigorous curricula, which include frequent theoretical and practical assessments (3, 6, 23, 24). A minimum GPA is required in dental education to demonstrate academic competence and preparedness for a demanding curriculum. Admissions committees utilize GPA as a key criterion to evaluate a candidate's potential for success in a program characterized by challenging coursework, and cumulative GPAs. A high GPA reflects effective time management, critical thinking, and study skills, all of which are essential for success in dental school and the profession.

This study was conducted to identify the factors associated with poor academic performance among dental students in Riyadh, Saudi Arabia. The research documented several factors that significantly impacted the academic performance of the participants. In the present study, gender played a crucial role, with male students being 2.1 times more likely to have a lower GPA compared to female students. Similar findings were reported in a study conducted by George B. et al., (3) where female dental students demonstrated better academic performance in all academic years compared to their male counterparts in India (3). Similar finding wwere also reported by Fernandez, M. dos S. et al. (17) who observed that female students demonstrated better academic performance compared to male dental students in Brazil (17). Gender differences have also been observed in the realm of emotional intelligence among dental students (25). Female dental students exhibit greater emotional sensitivity, motivation and expressiveness than their male counterparts (25–27). Erdinç G. and Tokgöz Kaplan T. (28) reported in their study that female dental students faced more stress compared to their male counterparts (28). In contrast, Jowkar Z. et al. (29) reported that there was no difference in dental environmental stress between female dental students. However, female students exhibited better academic performance compared to male dental students in Iran (29).These gender-related differences may also be attributed to students’ learning experiences, self-confidence, and preferred learning styles (30).

Motivation to continue the course was another strong predictor; students with higher motivation were less likely to have a lower GPA in this study. Research indicates that students who are motivated, inspired, and driven generally achieve superior outcomes (30). Research also indicates that as students’ progress through their academic years, there is a notable increase in academic motivation. This may be attributed to heightened academic demands and professional expectations (31, 32). Such changes likely reflect a stronger sense of purpose and a closer alignment with career objectives (19).

In the present study, language was another important factor with a significant impact on the students’ academic performance. English is used as a medium of instruction (EMI) and is increasingly prevalent in universities across Saudi Arabia (33). EMI refers to teaching academic subjects in English in environments where most students are native speakers of another language, specifically Arabic in Saudi Arabia (34). In healthcare education, EMI is often implemented to enhance students’ global competitiveness. However, pursuing health professional courses in English can present challenges for non-native speakers. This may lead to increased levels of stress, mental fatigue, and cognitive overload (34, 35). In the present study English language difficulties significantly impacted academic performance, with students perceiving English as a barrier being 2.8 times more likely to have a lower GPA. These findings were in accordance with the findings reported by another study conducted in Saudi Arabia suggesting that EMI can significantly contribute to student burnout in healthcare education (36). Without adequate language support and institutional adjustments, EMI may negatively affect students’ mental health, academic performance, and motivation (37).

In the present study, irregular attendance also emerged as a significant predictor, with students who frequently missed classes being 2.5 times more likely to have poor academic performance. These findings are consistent with those reported by Ait-Aissa, K. et al. (38) who found that attendance plays a critical role in determining grades independently (38). Absenteeism presents a significant challenge in health professions education, characterized by limited access to educational resources, disruptions in continuity, reduced engagement, and increased workload. These factors can negatively impact academic performance, highlighting the importance of consistent attendance for students to enhance their chances of achieving higher grades (38–40).

Another significant factor was sleep quality, which is frequently associated with students’ academic performance. Poor sleep quality has been linked to lower grades and increased rates of depressive symptoms among students (42). Higher stress levels have been observed in individuals experiencing poor sleep quality (42). Previous research has noted that dental students endure more stress compared to those in other health fields, such as medicine (43). In this study, sleep quality emerged as a crucial factor, with students reporting good sleep quality being less likely to have a low GPA. These findings align with those reported by Muñoz M.S. et al. (12) who observed a high prevalence of poor sleep quality associated with poorer academic performance among undergraduate dental students in Brazil (12). Another study by Fernandez, M. dos S. et al., (17) also reported that poor sleep quality had 3.1 times higher chance with poor academic performance in Brazilin dental students. Additionally, a study by Elagra M. et al. (44) indicated that poor sleep quality was linked to lower academic performance, particularly among dental students in their clinical years (44). In contrast, Jowkar Z. et al. (29) reported that they did not identify a significant relationship between sleep quality and academic performance among dental students in Iran (29).

Another noteworthy aspect was the time spent with family and friends, which influenced the participants’ academic performance. A meta-analysis indicated that social interactions with family and friends often create a supportive environment and promote positive behaviors, facilitating knowledge exchange and ultimately enhancing learning experiences (45, 46). However, in the current study, it was observed that the time devoted to social interactions with family and friends tended to increase the likelihood of poor academic performance, as students who engaged more in socializing were more prone to having a lower GPA. These findings align with previous studies that have indicated excessive socializing can become a distraction, leading to poor time management and a lack of focus on academic work, which can negatively affect a student's academic performance (47).

In the current study, smoking status was found to play a significant role, with smokers being more prevalent in the lower GPA group. These findings are consistent with previous research conducted among secondary school and medical and health sciences students in Saudi Arabia, which reported a significant association between smoking and poor academic performance (48–50). Furthermore, these studies highlighted the detrimental effects of smoking on cognitive functions such as memory and attention, which contribute to lower GPAs, increased absenteeism, and a higher number of academic warnings (49, 50). Smoking not only impacts academic performance but also adversely affects an individual's health and well-being. Recognizing the vital role dental professionals play in tobacco prevention and cessation, it is essential to develop and implement tobacco control programs specifically for dental students.

The current research has identified several critical factors significantly associated with inadequate academic performance. The results indicate that lack of motivation, inconsistent attendance, poor sleep quality, and insufficient proficiency in English, smoking, substantially impact the academic performance of dental students. Understanding the influence of these factors on poor academic performance can help the university develop strategies to enhance the educational experience in dental schools and implement appropriate interventions and support services for students. Within campus, it is essential to focus on recognizing and addressing students’ academic challenges, sociocultural adaptation, well-being, and other difficulties. Interventions designed to enhance students’ well-being, when implemented in university settings, can be more beneficial (51). Universities should aim to identify the academic struggles and needs of low-performing students at an early stage and support their education through targeted remediation and counseling services (52).

The current research has several limitations, one of which is its cross-sectional design. Data were collected at a single point in time, preventing the establishment of causal relationships. Future studies should employ a longitudinal design to address this limitation. Another limitation concerns questions about sleep quality, where responses were dichotomous and subjective, potentially leading to inaccurate estimations. Additionally, the reliance on self-reported data for variables such as attendance is susceptible to recall and social desirability biases. Another limitation was the reliance on self-reported GPAs by the participants, as official administrative records were not provided by the authorities of the participating universities. This may affect the internal validity of the findings due to the lack of official data.

## Conclusions

In the present study, several factors were found to affect the academic performance of dental students. Lack of motivation, inconsistent attendance, poor sleep quality, smoking, male gender, and insufficient proficiency in English significantly impact the academic performance of dental students. These findings provide a clear, evidence-based roadmap for dental colleges to develop targeted and effective interventions. By implementing robust language support systems, creating mentorship programs to ease the transition into clinical practice, and fostering a learning environment that enhances student motivation and well-being, institutions can better support their students, improve academic outcomes, and ultimately contribute to the development of a more competent future generation of dental professionals.

## Data Availability

The original contributions presented in the study are included in the article/Supplementary Material, further inquiries can be directed to the corresponding author.
